# Integrating QTL Mapping and Candidate-Region Association to Investigate Kernel Size and Weight in Sweet Corn

**DOI:** 10.3390/ijms27219863

**Published:** 2026-11-03

**Authors:** Chongjing Ma, Jinrong Song, Jingtao Qu, Hongjian Zheng, Tao Li, Shujun Li, Diansi Yu, Wei Gu, Hui Wang, Kai Li, Yingjie Zhao, Zhixiong Zhao, Felix San Vicente, Ying Xie, Yuan Guan

**Affiliations:** 1 CIMMYT-China Specialty Maize Research Center, Crop Breeding and Cultivation Research Institute, Shanghai Academy of Agricultural Sciences, Shanghai 201403, China; chongjing200106@163.com (C.M.); 18309319828@163.com (J.S.); qujingtao@saas.sh.cn (J.Q.); hjzh6188@163.com (H.Z.); miraitowa0914@163.com (T.L.); 15163870727@163.com (S.L.); yds.bio@hotmail.com (D.Y.); guwei@saas.sh.cn (W.G.); wanghui19840109@163.com (H.W.); likai@saas.sh.cn (K.L.); zhaoyingjie@saas.sh.cn (Y.Z.); 13645518440@163.com (Z.Z.); fsanvi60@gmail.com (F.S.V.); 2 College of Agronomy, Sichuan Agricultural University, Chengdu 611130, China

**Keywords:** sweet corn, kernel size, kernel weight, quantitative trait loci, candidate genes

## Abstract

Sweet corn is widely cultivated for fresh consumption and processing, and kernel size and weight are important yield-related traits. However, the genetic architecture of these traits in sweet corn remains poorly understood. In this study, the SHL01 × SHL03 recombinant inbred line (RIL) population was used to investigate the genetic basis of kernel size and weight-related traits using a high-density SNP-based genetic map. Quantitative trait loci (QTL) mapping was conducted in two environments and using the best linear unbiased estimates (BLUEs), followed by candidate-region association analysis to identify candidate genes. A total of 11 unique QTLs were identified from 12 detection records across the two environments and the BLUE dataset. Among them, seven unique QTLs, corresponding to eight detection records, were located on chromosome 7. Several QTLs were located near previously reported loci for kernel-related traits, suggesting a partially shared genetic basis between sweet corn and field corn. Candidate-region association analysis further characterized the major QTL regions and highlighted five potential candidate genes. Among them, *Zm00001d020325* (*HAK23*), encoding a putative potassium transporter, was supported by functional annotation and available expression information, but its haplotypes were not significantly associated with HKW. In contrast, haplotypes defined by variants in neighboring *Zm00001d020323* differed in HKW, with the GG haplotype showing the highest mean value. Because these analyses were conducted in overlapping populations, the haplotype results are exploratory. Together, these findings characterize genomic regions associated with mature-kernel traits in sweet corn and identify candidates for further validation.

## 1. Introduction

Sweet corn (*Zea mays* L.) is widely cultivated worldwide and is an important vegetable crop in many countries such as China, the United States, and Canada. It provides dietary fiber, folate, carotenoids, and other nutrients [1]. In recent years, with the expansion of sweet corn production, its utilization has gradually extended beyond traditional fresh consumption to a range of processed products, including frozen kernels, canned corn, vacuum-packed ears, and corn-based beverages [2]. According to incomplete industry statistics, approximately 0.5 million tons of sweet corn kernels are processed annually in China, indicating substantial demand for sweet corn as a processing raw material [3]. Thus, sweet corn production needs not only meet the demands of the fresh market for flavor and eating quality but also provide a stable supply of raw materials for processing industries. Consequently, improving yield is important for meeting the growing demand for sweet corn from both fresh and processing markets. However, yield is a complex quantitative trait controlled by multiple genetic loci and strongly influenced by environmental conditions [4]. Kernel length (KL), kernel width (KW), and hundred-kernel weight (HKW) are important yield-related component traits that reflect aspects of kernel growth and size determination. Although sweet corn is generally harvested at the immature stage for fresh consumption, these traits measured in mature kernels can provide information on the genetic factors underlying kernel development and weight formation. They are therefore relevant to investigating the genetic basis of kernel-related traits, although they do not directly represent kernel dimensions, fresh weight, or eating quality at the fresh-harvest stage. The heritability of these traits varies with genetic background and experimental conditions, and their expression may also be affected by environmental factors and genotype × environment (G × E) interactions [5]. Therefore, identifying genetic loci associated with kernel size and weight in sweet corn and characterizing their potential candidate genes are important for understanding the genetic basis of yield-related traits and supporting marker-assisted breeding.

Quantitative trait loci (QTL) mapping has been widely used to dissect the genetic basis of important agronomic traits in maize. Using different types of genetic populations and mapping approaches, previous studies have identified numerous QTLs associated with KL, KW, and HKW, although the proportion of phenotypic variation explained (PVE) by individual QTLs varies considerably among studies [6,7,8,9,10]. For example, a study involving 11 recombinant inbred line (RIL) populations derived from crosses between Huangzao4 and 11 maize inbred lines identified 35 QTLs for KL, 43 for KW, and 26 for HKW across multiple environments, with some QTLs explaining more than 10% of the phenotypic variation [8]. Previous studies have also reported major-effect QTLs for kernel size and weight. For instance, *qKWid9* explained 13.4% of the phenotypic variation in KW [7], while the PVE of some KL-related QTLs reached 15.3% [10]. These findings indicate that kernel-related traits in maize are controlled by multiple genetic loci, some of which have relatively large genetic effects. Although some QTLs have been repeatedly detected across populations or environments, the reproducibility of individual loci is not guaranteed. QTL detection and estimated genetic effects may vary among studies because of differences in genetic background, population type and size, environmental conditions, and experimental or statistical procedures. Marker density and mapping resolution should also be distinguished. Adequate single-nucleotide polymorphism (SNP) marker coverage facilitates genotype characterization and the identification of recombination breakpoints, but increasing marker density alone does not necessarily improve the precision of QTL localization. In biparental populations, mapping resolution is also constrained by population size and the number and distribution of informative recombination events. Consequently, QTL intervals may remain relatively broad even in densely genotyped populations, complicating the fine mapping of QTLs and the identification of candidate genes [11].

Association analysis based on diverse natural populations can take advantage of historical recombination accumulated during long-term evolution and therefore may provide higher mapping resolution than conventional linkage mapping [12]. However, population structure and genetic relatedness may influence association results, while linkage disequilibrium (LD) affects the resolution and interpretation of significant association signals. Therefore, these factors should be considered when interpreting regional association results. Regional association analysis focuses on specific genomic regions identified through linkage mapping and further exploits recombination information within these regions to identify candidate loci [13]. This approach combines the detection capability of linkage mapping with the potential higher resolution of association analysis [14], helping to narrow candidate intervals and identify potential candidate genes. In recent years, this strategy has been applied to dissect the genetic basis of important agronomic traits in maize, including kernel test weight, ear-related traits, and kernel proportion [15,16,17,18]. Although considerable progress has been made in the genetic dissection of kernel-related traits in maize, existing studies have focused predominantly on field maize, while the corresponding studies in sweet corn remain relatively limited. Due to the effects of endosperm mutant genes, alterations in carbohydrate metabolism and starch accumulation can affect endosperm development and kernel filling, resulting in the wrinkled and irregular appearance of mature sweet corn kernels [19]. These characteristics not only increase the difficulty of phenotypic evaluation but also complicate the dissection of their genetic basis. Therefore, integrating linkage mapping with regional association analysis to further characterize loci associated with kernel size and weight in sweet corn may help refine major QTL regions and facilitate candidate gene identification.

Although previous studies have identified numerous QTLs for kernel-related traits in maize, the genetic basis of kernel size and weight in sweet corn remains insufficiently understood, particularly the identification of candidate genes within major QTL regions. In this study, we constructed an RIL population using the inbred sweet corn lines SHL01 and SHL03 and evaluated KL, KW, and HKW in two environments. QTL mapping was first performed to identify genomic regions associated with these traits. Candidate-region association analysis was then conducted within the selected QTL intervals to characterize local association signals and prioritize potential candidate genes. Haplotype analysis was subsequently performed for selected candidate genes to examine their associations with kernel traits. This study aimed to improve our understanding of the genetic basis of kernel size and weight in sweet corn, provide additional evidence for candidate gene identification within major QTL regions, and provide valuable information for molecular marker-assisted breeding of sweet corn.

## 2. Results

### 2.1. Phenotypic Analysis

**Figure 1:**
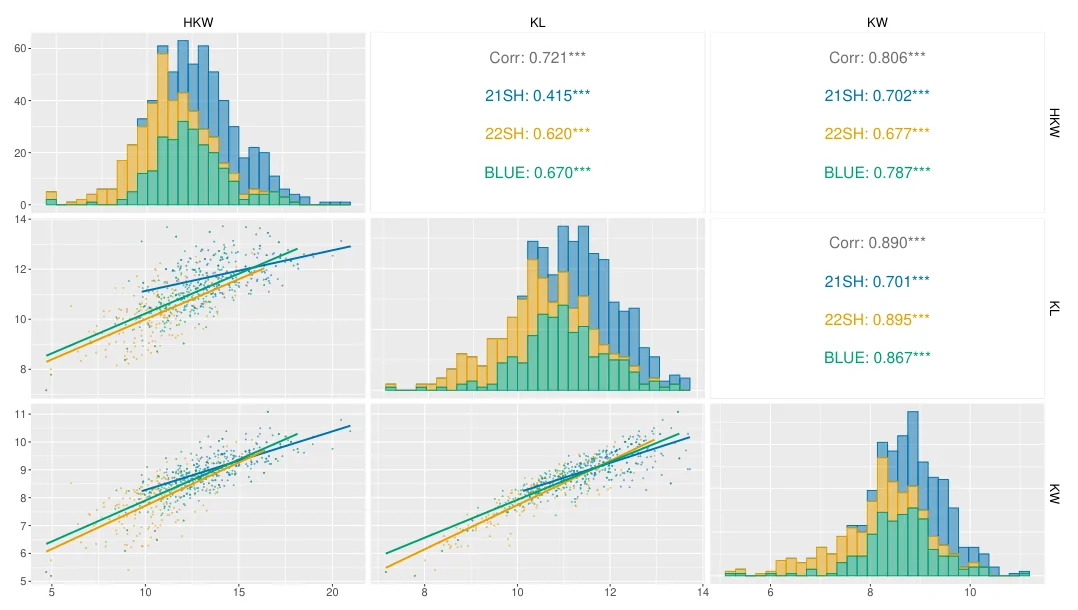
Correlation analysis and phenotypic distribution of kernel-related traits in the RIL population across different environments and BLUE values. Pearson correlation coefficients are shown in the upper panels, histograms in the diagonal panels, and scatterplots with fitted regression lines in the lower panels. Overall correlations are shown in gray, while environment-specific results are shown in blue (21SH), orange (22SH), and green (BLUE). HKW, hundred-kernel weight; KL, kernel length; KW, kernel width. 21SH, Shanghai in 2021; 22SH, Shanghai in 2022; BLUE, best linear unbiased estimate. *** indicates significance at p<0.001.

The parental lines, SHL01 and SHL03, exhibited differences in kernel-related traits, and the derived RIL population showed extensive phenotypic variation for all investigated traits across the two environments (21SH and 22SH). As shown in Table 1, the mean values of all three traits were lower in 22SH than in 21SH. The most pronounced difference was observed for HKW, which decreased from 14.08 g to 10.44 g, while the mean values of KL and KW decreased from 11.77 mm to 10.14 mm and from 9.12 mm to 7.85 mm, respectively. The phenotypic ranges also differed between environments, with coefficients of variation (CVs) ranging from 6.58% to 19.35%. HKW showed greater relative variation than KL and KW in both environments. Skewness and kurtosis values ranged from −0.43 to 0.61, suggesting relatively limited deviations from normality. The Q-Q plots also indicated that the phenotypic distributions were generally close to normal, although slight deviations were observed at the distribution tails (Figure S1). Analysis of variance (ANOVA) revealed significant effects of genotype and genotype × environment interaction for HKW, KL, and KW (p<0.01), indicating genetic differences among lines and environment-dependent variation in their phenotypic responses. Broad-sense heritability ($H^{2}$) ranged from 0.53 to 0.64. Best linear unbiased estimates (BLUEs) were used to summarize genotype performance across the two environments; however, given the significant genotype × environment interactions, these estimates should not be interpreted as evidence of consistent performance across environments. Pearson correlation analysis revealed significant positive correlations among all three kernel-related traits in both environments and in the BLUE dataset (Figure 1). KL and KW showed the strongest correlation (r=0.890), while HKW was positively correlated with KL and KW (r=0.721 and 0.806, respectively). The positive correlations were generally consistent across the evaluated datasets, suggesting that larger kernels tended to have higher kernel weight.

### 2.2. QTL Mapping of Kernel Traits

**Figure 2:**
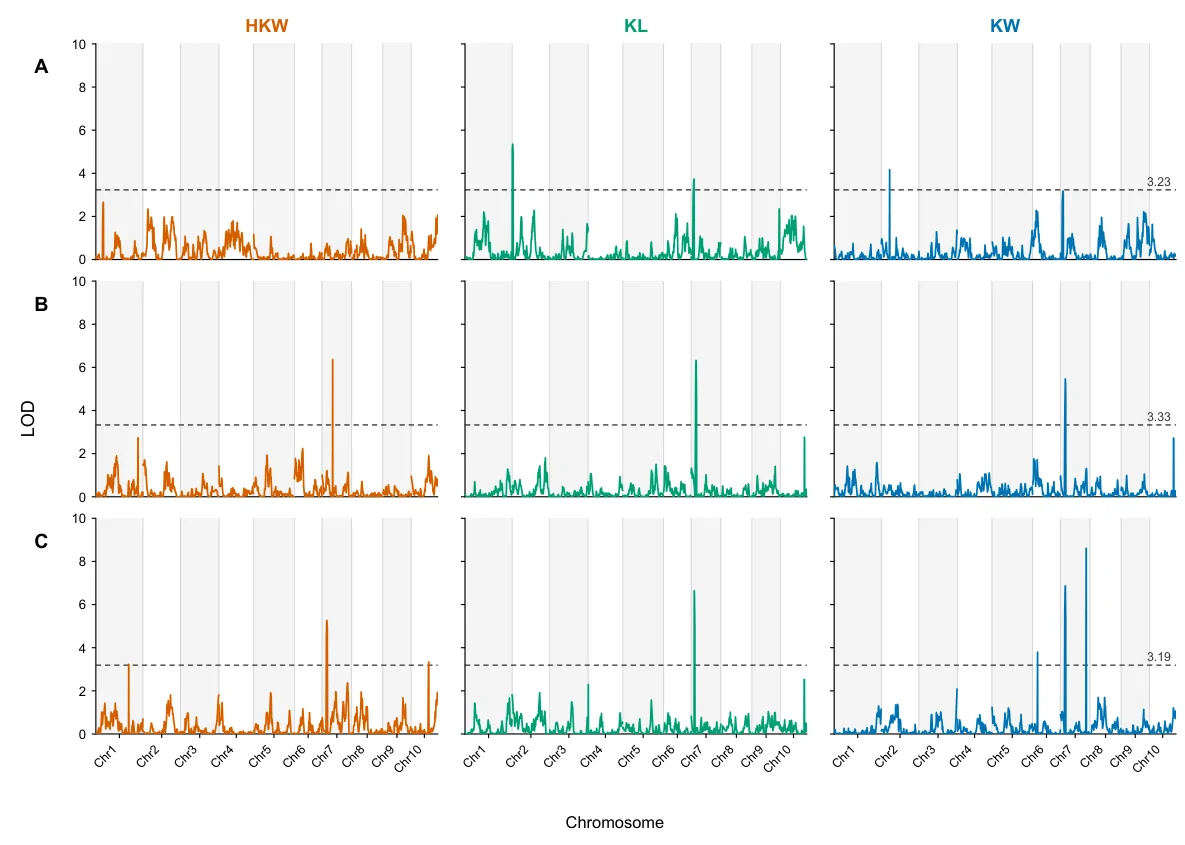
Genome-wide QTL profiles for grain-related traits across two environments and the BLUE dataset in the RIL population. The dashed horizontal lines indicate the LOD thresholds determined by 1000 permutation tests at a genome-wide significance level of α=0.05 (21SH: 3.23; 22SH: 3.33; BLUE: 3.19). (**A**) QTL mapping under 21SH environment; (**B**) QTL mapping under 22SH environment; (**C**) QTL mapping based on BLUE values across environments.

**Figure 3:**
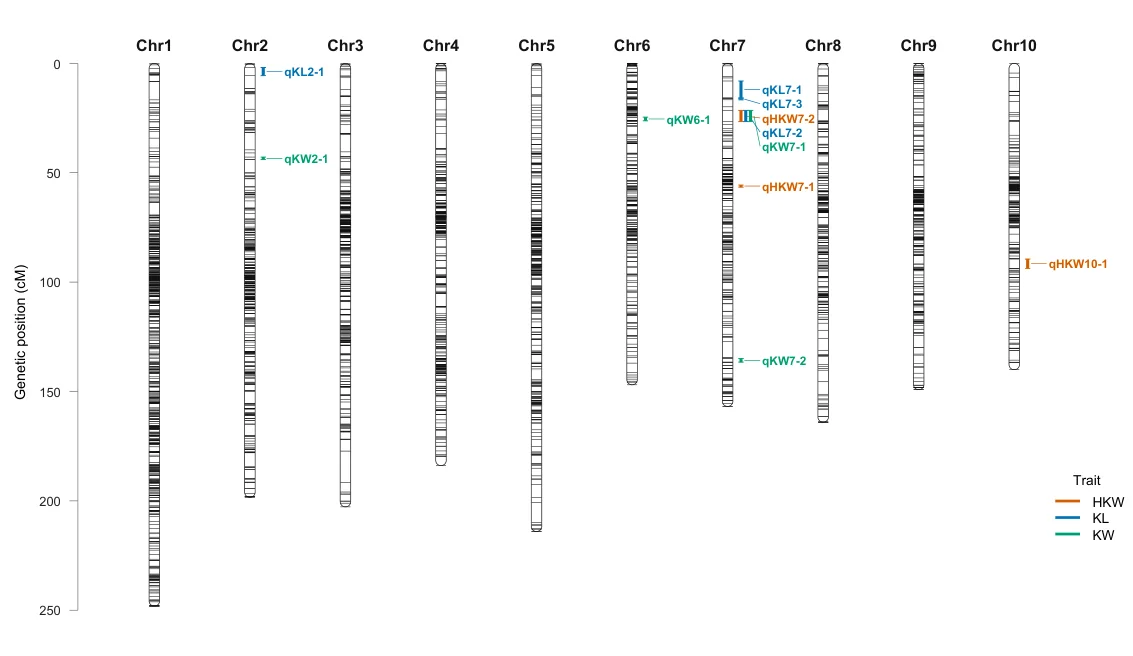
Distributions of identified QTLs for three kernel-related traits on genetic linkage maps. QTLs were named as “q + trait abbreviation + chromosome number + locus number”. Black, gray, and white bands indicate the distribution of missing genotype data across the linkage groups.

Genotyping of the RIL population using genotyping by target sequencing (GBTS) yielded 4199 high-quality SNP markers after quality filtering, of which 4195 were mapped to the 10 maize chromosomes. The genetic linkage map spanned 1804.11 cM, with an average interval of 0.43 cM between mapped markers and a maximum gap of 14.41 cM. The mapped markers occupied 1962 distinct genetic positions. QTL mapping was subsequently performed using the ICIM method with phenotypic data collected from the 21SH and 22SH environments as well as BLUE values across environments (Table S1). A total of 12 QTL detections, corresponding to 11 unique QTLs, were identified across the 21SH, 22SH, and BLUE datasets (Figure 2). The distribution of SNP markers and the identified QTLs on the genetic linkage map is shown in Figure 3.

The QTLs were distributed across chromosomes 2, 6, 7, and 10, including four QTLs for KL, four for KW, and three for HKW. Among them, *qKW7-1* was detected in both 22SH and the BLUE dataset and was counted as a single QTL because the two detections shared the same genomic interval. Logarithm-of-odds (LOD) scores ranged from 3.34 to 8.62, with individual QTLs explaining 4.04–13.90% of the phenotypic variation (Table 2). In the 21SH environment, 3 QTLs were detected, including 2 KL-related QTLs and 1 KW-related QTL located on chromosomes 2 and 7. Among them, *qKL2-1* and *qKW2-1* explained 10.78% and 8.26% of the phenotypic variation, respectively. In the 22SH environment, 3 QTLs associated with HKW, KL and KW were identified, all located on chromosome 7. Notably, *qKL7-2* and *qKW7-1* explained 13.90% and 13.10% of the phenotypic variation, respectively, representing major-effect QTLs detected in this study. Based on BLUE values, 6 QTLs were identified, including *qHKW7-2*, *qHKW10-1*, *qKL7-3*, *qKW6-1*, *qKW7-1*, and *qKW7-2*. Among them, *qKW7-2* exhibited the highest LOD score (8.62), while *qKL7-3* explained 13.40% of the phenotypic variation. Chromosome 7 contained the largest number of QTL detections, with eight detection records corresponding to seven unique QTLs. Among these, the 4.8–5.3 Mb interval was associated with KL and KW in 22SH and with HKW and KW in the BLUE analysis, suggesting that this region may contribute to variation in multiple kernel-related traits.

### 2.3. Candidate Gene Identification by Integrating QTL Mapping and Candidate-Region Association Analysis

To further investigate QTLs with relatively large phenotypic effects, five QTLs with PVE greater than 10% were selected for regional association analysis, including *qKL2-1*, *qKL7-2*, *qKL7-3*, *qKW7-1*, and *qHKW7-1*. This threshold was adopted as a practical criterion to prioritize QTLs explaining a relatively large proportion of phenotypic variation for further analysis. Among these QTLs, *qKL7-2* and *qKW7-1* shared the same physical interval, resulting in four distinct genomic regions for subsequent analysis. Based on the B73 RefGen_v4 reference genome assembly, the five QTL intervals were located in Chr2 (1,662,571–1,819,689 bp), Chr7 (4,744,844–4,783,865 bp), Chr7 (3,320,507–3,343,680 bp), Chr7 (4,744,844–4,783,865 bp) and Chr7 (106,544,511–106,767,536 bp), respectively. Re-screening of the original resequencing data identified 882, 373, 346, 373, and 271 high-quality SNP markers within these intervals, respectively (Table S2 and Table S3). For each QTL region, regional association analysis was performed using SNP markers within the corresponding interval and a linear mixed model. A suggestive threshold of p<1/n was used to screen SNP–trait associations, where *n* represents the number of SNP markers tested within each region. A total of 66 suggestive SNP–trait association records were identified across the five QTL analyses (Table S3; Figure S2). After multiple-testing correction across the 2245 SNP–trait tests, 49 association records remained significant under the BH-FDR threshold of 0.05, whereas none passed the Bonferroni correction. Therefore, these associations were considered exploratory evidence for candidate-gene prioritization.

LD patterns were further examined around the selected association signals to identify potential candidate genes (Figure S3). Based on the suggestive association signals, local LD patterns, and available gene annotations, five potential candidate genes were prioritized. These included *Zm00001d001845*, *Zm00001d018770*, and *Zm00001d018732* within the *qKL2-1*, *qKL7-2*, and *qKL7-3* regions, respectively, and *Zm00001d020323* and *Zm00001d020325* within the *qHKW7-1* region. Additional annotated genes within the selected candidate regions are provided in Table S4. Although *qKW7-1* and *qKL7-2* shared the same physical interval, the association analysis for KW did not identify a clear candidate-gene-associated LD pattern. The functional annotation of the candidate genes showed that *Zm00001d018732* encodes a Chaperone DnaJ-domain superfamily protein, *Zm00001d018770* encodes a GYF domain-containing protein, and *Zm00001d020325* encodes the potassium high-affinity transporter (*HAK23*). *Zm00001d001845* and *Zm00001d020323* were annotated as genes with unknown functions (Table 3). To further evaluate population structure, PCA was performed using 82,657 LD-pruned genome-wide SNP markers from the 150 association lines. PC1, PC2, and PC3 explained 8.45%, 4.22%, and 3.28% of the total genetic variation, respectively (Figure S4). The effects of genetic relatedness and population structure on regional association results were further evaluated by comparing the original region-specific kinship model with LOCO kinship models with and without the first three principal components (Figure S5). No SNPs remained significant after BH-FDR or Bonferroni correction under either LOCO model. These results indicate that the regional association signals were sensitive to kinship model specification.

### 2.4. Expression Patterns of Candidate Genes During Embryo and Endosperm Development

Publicly available transcriptomic data from the field corn inbred line B73 (E-MTAB-4395) were used to analyze the expression profiles of candidate genes in different developmental stages. This dataset systematically profiled embryo and endosperm tissues at different stages of kernel development and generated gene expression data using RNA-seq (Table S5), providing an additional expression-based assessment of the potential association between the candidate genes and kernel development.

**Figure 4:**
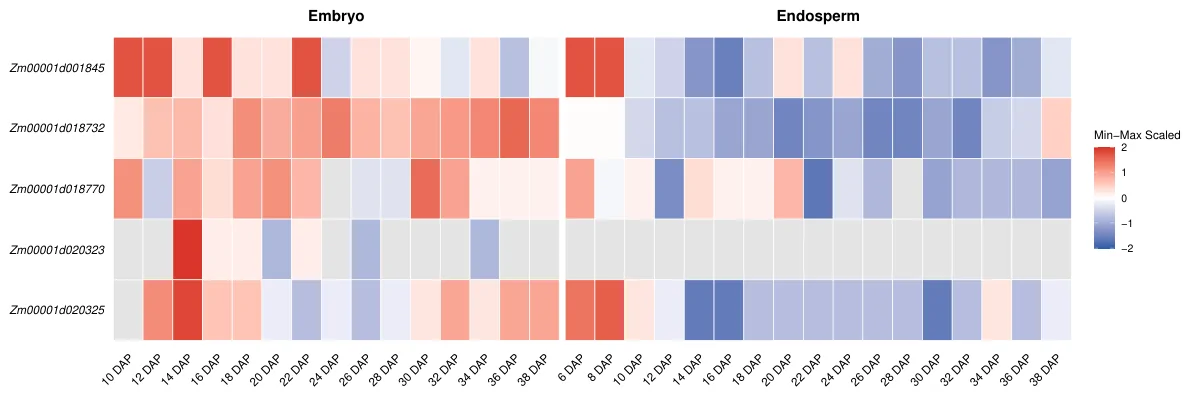
Expression patterns of candidate genes across maize embryo and endosperm. Transcript abundance was derived from published RNA-seq datasets [20] (E-MTAB-4395). FPKM values were log_2_-transformed, and row-wise min–max scaling was applied to a range of −2 to 2 for visualization. Red and blue colors indicate relatively higher and lower expression levels within each gene’s developmental trajectory, respectively. This scaled heatmap is intended to show relative temporal trends and does not represent absolute transcript abundance across different genes. Gray cells indicate unavailable expression values.

As shown in Figure 4, the five candidate genes exhibited distinct temporal expression patterns in embryos and endosperms. *Zm00001d001845* showed relatively high expression in embryos during the early developmental stages, whereas its expression in endosperms decreased from relatively high levels at early stages to relatively lower levels at later stages. *Zm00001d018732* showed relatively sustained high expression in embryos and maintained a relatively high level during later development, while its expression in endosperms was generally lower. *Zm00001d018770* showed developmental variation in both embryo and endosperm tissues. *Zm00001d020325* exhibited changes in embryo expression, with relatively high expression during early development, followed by a decrease at intermediate stages and a subsequent increase. Its expression in endosperms also varied across developmental stages. In contrast, *Zm00001d020323* showed very low expression levels (FPKM = 0.1–0.4) at only seven of the 15 examined embryo stages, whereas expression values were unavailable for the remaining eight embryo stages and all 17 endosperm stages (Table S5). Overall, the available transcriptomic data showed developmental variation in the expression of four candidate genes in B73 embryos and endosperms. However, the limited expression data for *Zm00001d020323* did not allow a reliable assessment of its developmental expression pattern. These results provide additional information on the expression of candidate genes during kernel development but do not directly establish their roles in regulating kernel size or weight in sweet corn.

### 2.5. Haplotype Analysis and Identification of a Favorable Haplotype

Based on its functional annotation and available transcriptomic information, *Zm00001d 020325* (*HAK23*) was selected for haplotype analysis. However, no significant association with HKW was detected for this gene in the present population. Given its close proximity to *Zm00001d020323*, we extended the haplotype analysis to this neighboring gene. A total of 23 polymorphic markers were identified in *Zm00001d020323* and its flanking 2-kb regions. Three markers showed significant associations with HKW after Bonferroni correction. Comparison of phenotypic differences among genotype classes resulted in the selection of two SNPs, 7:106546133 G>A and 7:106546304 G>C, which showed clear differences in phenotypic values and were used for haplotype construction.

**Figure 5:**
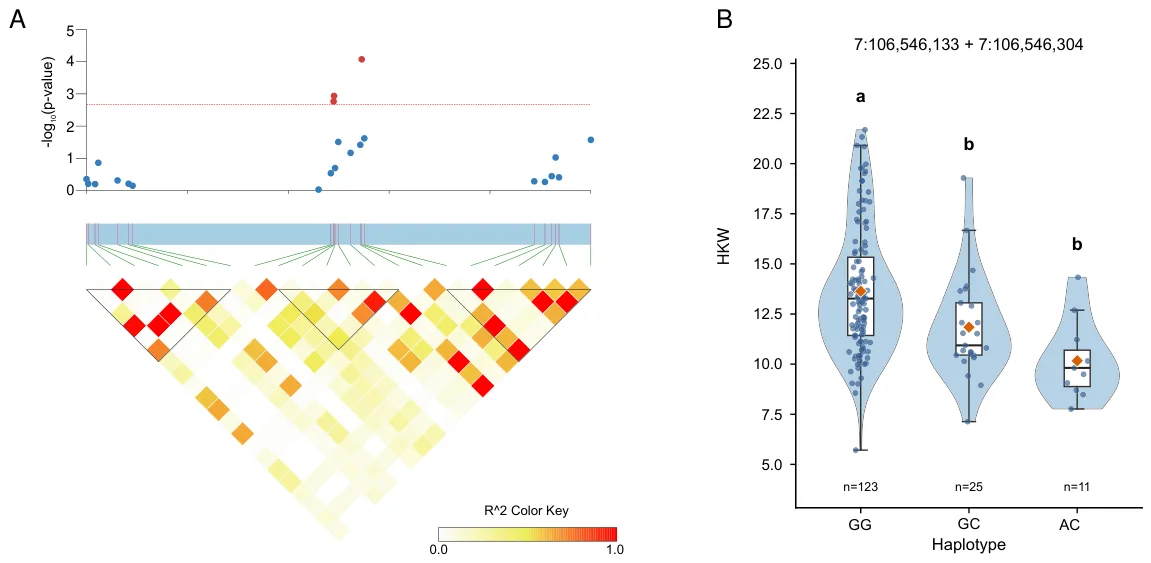
Haplotype analysis of *Zm00001d020323* and its association with hundred-kernel weight (HKW). Haplotype groups included GG (n=123), GC (n=25), and AC (n=11), totaling 159 lines with available HKW phenotypic data. (**A**) Regional association signals (top), SNP positions (middle), and pairwise LD patterns (bottom). The red dashed line indicates the Bonferroni-corrected threshold (p=0.05/23); red points indicate SNPs passing this threshold, and blue points indicate SNPs not passing it. Green lines link SNP positions to the corresponding entries in the LD heatmap. The LD heatmap shows pairwise $r^{2}$ values according to the color scale. (**B**) Violin plots show HKW distributions among the three haplotypes; internal boxplots show the median and interquartile range, points represent individual lines, orange diamonds indicate group means, and different letters indicate significant differences among haplotypes (p<0.05).

The two polymorphic sites defined three major haplotypes, namely GG, GC, and AC (Figure 5). Significant differences in HKW were observed among the three haplotypes (one-way ANOVA, *F* = 9.45, *p* = 1.33 × 10^−4^; Kruskal–Wallis test, *H* = 20.59, *p* = 3.37 × 10^−5^). The GG haplotype showed the highest mean HKW (13.64 ± 3.15), which was significantly higher than those of the GC (11.84 ± 2.54) and AC (10.17 ± 1.93) haplotypes, whereas no significant difference was detected between the GC and AC haplotypes. These results indicate that the GG haplotype was associated with higher HKW and may represent a favorable haplotype for kernel weight improvement. However, as the SNPs used for haplotype construction were selected based on phenotypic associations in a largely overlapping population, these findings should be considered exploratory rather than independent validation of *Zm00001d020323*.

## 3. Discussion

### 3.1. Genetic Architecture of Kernel-Related Traits in Sweet Corn

Integrating QTL mapping, candidate-region association analysis, transcriptomic expression profiles, and haplotype analysis provides complementary evidence for prioritizing candidate genes and elucidating the genetic basis of kernel-related traits in sweet corn. Kernel size and weight are complex yield-related traits in maize, but their genetic basis has been studied predominantly in field corn [21]. In contrast, differences in endosperm sugar metabolism and starch accumulation in sweet corn result in distinct kernel development compared with field corn, and the genetic regulation of kernel-related traits in sweet corn remains insufficiently understood. In the present study, a high-density SNP-based linkage map was used to investigate the genetic basis of kernel size and weight-related traits in a sweet corn RIL population. Across the three datasets (21SH, 22SH, and BLUE), 11 QTLs associated with HKW, KL, and KW were identified on chromosomes 2, 6, 7, and 10. It should be noted that the estimated PVE of significant QTLs may be upwardly biased because QTL detection and effect estimation were performed using the same population. Therefore, these estimates require further evaluation in independent populations. Previous studies have shown that QTLs and candidate genes associated with maize kernel development can cluster within specific chromosomal regions or QTL hotspots, where genetic factors controlling kernel size and weight may be enriched [22]. The concentration of QTLs on chromosome 7 suggests that this chromosome may contain important genomic regions associated with kernel-related traits in sweet corn. Notably, several QTLs were located within overlapping intervals, which may reflect pleiotropic effects or closely linked loci rather than independent genetic factors. However, these possibilities cannot be distinguished based on the current mapping resolution, and further investigation is needed to clarify the genetic basis of this clustering.

### 3.2. Comparison with Reported Kernel-Related QTLs

An important consideration in evaluating the accuracy of the QTLs identified in the present study is whether kernel-related QTLs previously reported in field corn are also detectable in sweet corn. The *qKL2-1* corresponded to a region identified in a previous multi-environment QTL mapping study using an F2 population derived from SG5/SG7 [10], while the interval identified in the present study was narrower. The *qKW2-1* was also located within the same chromosomal region reported in previous studies [10,23]. The *qHKW7-1* was located within a relatively large QTL interval of approximately 100 cM previously identified using SSR markers [24], whereas *qHKW10-1* corresponded to a previously reported QTL region for hundred-kernel weight [25]. These findings suggest that some genomic regions associated with kernel size and weight may be shared between sweet corn and field corn, despite differences in their genetic backgrounds and kernel characteristics. However, several QTLs identified in this study did not fully overlap with previously reported loci. Such differences may be attributed to variations in genetic background, segregating alleles, environmental conditions, and mapping resolution among studies. In addition, differences in kernel development and composition between sweet corn and field corn may contribute to variation in the detectable genetic effects. Therefore, the incomplete overlap of QTL regions does not necessarily indicate distinct genetic loci, and further investigation is needed to determine whether these regions contain previously unreported genetic variants.

### 3.3. Environmental Variation and Co-Localization of Kernel-Related QTLs

The mean values of HKW, KL, and KW were lower in 22SH than in 21SH. As the trials were conducted at the same location in different years, these differences may have been influenced by variation in seasonal growing conditions, although the contribution of individual environmental factors could not be determined from the available data. QTL detection also differed between the two field years. For example, *qKW7-1* was detected in 22SH and the BLUE dataset, but not in 21SH. Given the significant G×E interaction observed for KW, this difference may reflect variation in genetic effects across years, although differences in statistical power cannot be excluded [26]. Because QTL-by-environment interactions were not formally evaluated, the environmental stability of individual loci remains uncertain. In addition, *qKW7-1*, *qKL7-2*, and *qHKW7-2* co-localized within the 4.8–5.3 Mb region of chromosome 7, suggesting that this region may contribute to variation in multiple kernel-related traits. Similar clustering of kernel-related QTLs has been reported in maize [27]. However, whether these overlapping QTLs reflect pleiotropy or closely linked loci remains unresolved.

### 3.4. Candidate Genes Potentially Regulating Kernel Development

RIL populations are useful for identifying QTLs associated with maize kernel traits; however, the limited number of recombination events generally restricts mapping resolution. To further investigate the major QTL regions, candidate-region association analysis was conducted using a natural population of 150 fresh edible sweet corn lines [28], leading to the identification of five candidate genes. Among them, *Zm00001d020325* (*HAK23*) encodes a putative high-affinity potassium transporter belonging to the HAK/KUP/KT family [29]. Previous studies have reported its expression in developing embryos and endosperms [30]. In addition, a multi-locus GWAS reported an association between the corresponding locus and ear length general combining ability (GCA) [31]. Although this finding does not directly establish a role for *HAK23* in kernel size or weight, it provides related genetic context for further investigation. Analysis of publicly available B73 kernel developmental transcriptome data revealed developmental expression patterns for several candidate genes [20]. However, *Zm00001d020323* exhibited very low expression levels at a limited number of embryo stages, with no available expression values for the examined endosperm stages. Therefore, the transcriptomic data provide limited support for this gene, and its potential association with kernel weight is primarily supported by the regional association and exploratory haplotype analyses rather than its expression profile.

Haplotype analysis provided further genetic evidence for the region but did not establish a direct association between *HAK23* haplotypes and HKW in the present population. In contrast, two polymorphic sites in or near the neighboring gene *Zm00001d020323* defined three major haplotypes that differed significantly in HKW, with the GG haplotype showing the highest mean value. This association supports a relationship between genetic variation in this region and kernel weight, although the responsible variant and gene remain unresolved. Taken together, QTL mapping, candidate-region association analysis, functional annotation, and developmental expression patterns highlight this region for further investigation. *Zm00001d020325* (*HAK23*) remains a biological candidate based on its annotation and expression evidence, whereas the haplotype results provide more direct evidence of an association between variation in or near *Zm00001d020323* and HKW. Although 49 SNP–trait association records remained significant after BH-FDR correction under the original region-specific kinship model, none remained significant under the LOCO models. These results highlight the sensitivity of regional association signals to kinship model specification and warrant cautious interpretation of the prioritized candidate genes. Further fine mapping and functional validation are needed to determine the genes and variants underlying the observed QTL effects.

### 3.5. Implications for Fresh Corn Molecular Breeding

The QTL regions and candidate genes identified in this study may provide potential targets for further genetic investigation and marker-assisted breeding in sweet corn [32]. The genomic regions covered by these QTLs and their annotated genes may also provide important entry points for further investigation of the regulatory networks underlying kernel development [33,34]. Future studies combining gene expression analysis, genetic transformation, and gene editing will be needed to further validate the biological functions of candidate genes [35,36,37], thus providing a theoretical basis and marker resources for molecular breeding in sweet corn.

## 4. Materials and Methods

### 4.1. Plant Materials, Field Planting, and Phenotyping

In the study, an RIL population consisting of 230 F_7_ lines was developed from a cross between the inbred sweet corn lines SHL01 and SHL03 using the single-seed descent (SSD) method. This population was used for genetic linkage mapping and QTL analysis. The population was planted in the field at Shanghai Academy of Agricultural Sciences’ experimental stations of Zhuanghang (Fengxian District, Shanghai) in 2021 (21SH) and 2022 (22SH). All field trials were conducted using a randomized complete block design with three replications at each location. Each line was planted in two rows, with 10 plants per row and a row length of 2.5 m. After self-pollination, five plants were retained for evaluation. The harvested ears were air-dried until the kernel moisture content was below 14%. Three ears were selected and shelled separately. Fifty kernels were randomly sampled from each ear to measure KL, KW, total kernel number (TKN), and total kernel weight (TKW) using an SC-G automatic seed testing system (WSeen, Hangzhou, China; http://www.wseen.com/ProductDetail.aspx?id=16&classid=28 (accessed on 29 October 2026)). HKW was calculated as follows:

(1) $$\mathrm{HKW}=\frac{\mathrm{TKW}}{\mathrm{TKN}}\times 100$$

### 4.2. Statistical Analysis of Phenotypic Data

Combined ANOVA, broad-sense heritability ($H^{2}$), and BLUEs for HKW, KL, and KW were calculated using the AOV module of QTL IciMapping software (version 4.2). Phenotypic data from the two experimental years (21SH and 22SH) were analyzed using the following model:

(2) $$Y_{ijk}=\mu +G_{i}+E_{j}+R_{k(j)}+{\left(GE\right)}_{ij}+\varepsilon_{ijk}$$

where $Y_{ijk}$ represents the phenotypic observation of the *i*-th genotype in the *j*-th year and *k*-th replication; μ is the overall mean; $G_{i}$ is the genotype effect; $E_{j}$ is the year effect; $R_{k(j)}$ is the replication effect nested within year; ${\left(GE\right)}_{ij}$ is the genotype-by-year interaction effect; and $\varepsilon_{ijk}$ is the residual error. For variance component estimation, year was treated as a fixed effect, whereas genotype, replication nested within year, and genotype-by-year interaction were treated as random effects.

Broad-sense heritability ($H^{2}$) was estimated on a genotype-mean basis as follows:

(3) $$H^{2}=\frac{\sigma_{G}^{2}}{\sigma_{G}^{2}+\frac{\sigma_{GE}^{2}}{e}+\frac{\sigma_{\varepsilon }^{2}}{er}}$$

where $\sigma_{G}^{2}$, $\sigma_{GE}^{2}$, and $\sigma_{\varepsilon }^{2}$ represent the genotypic, genotype-by-year interaction, and residual variance components, respectively; *e* is the number of years (2) and *r* is the number of replications per year (3). For BLUE estimation, genotype and year were treated as fixed effects, while replication nested within year and genotype-by-year interaction were treated as random effects. The resulting BLUE values were used for subsequent QTL mapping.

### 4.3. Genotyping of the RIL Population

The RIL population was genotyped using a maize 10K SNP marker panel based on GBTS technology. Young leaf tissues were collected from each of the 230 RILs, and genomic DNA was extracted using a modified CTAB method. The extracted DNA was used for library construction and target enrichment through probe hybridization. The enriched libraries were subsequently sequenced using paired-end 150-bp reads (PE150) on the MGISEQ-2000 platform (MGI, Shenzhen, China). Raw sequencing reads were processed using Fastp (v0.20.1) with the parameters “-q 20 - -length_required 50” to remove low-quality sequences and short reads. The filtered reads were aligned to the SHL01 reference genome using BWA-MEM (v0.7.17) with the “-M” option. The alignment files were sorted using SAMtools (v1.6), and duplicate reads were marked using Picard (v2.18.29). SNP calling was performed using FreeBayes (v1.3.6) with default parameters. The genotype dataset corresponding to the 10K SNP panel was generated using a Perl script developed by Mol Breeding Biotechnology Co., Ltd. (Shijiazhuang, China). The resulting SNP genotype data were used for linkage map construction after marker quality filtering, as described in Section 4.4. Physical positions of the SNP markers used for QTL mapping were based on the SHL01 reference genome.

### 4.4. Linkage Mapping

Linkage analysis was conducted using the SHL01/SHL03 RIL population. SNP markers with missing rates > 20%, heterozygosity rates > 20%, minor allele frequencies (MAF) < 0.05, or more than two alleles were excluded. After quality filtering, 4199 SNP markers were retained, of which 4195 were mapped to the 10 linkage groups using QTL IciMapping with the Kosambi mapping function [38]. Markers sharing the same genetic position were identified to describe marker redundancy.

Based on the genetic linkage map, QTLs underlying HKW, KL, and KW were identified using the inclusive composite interval mapping of additive QTLs (ICIM-ADD) method implemented in QTL IciMapping software [39]. QTL mapping was performed separately using phenotypic data from the 21SH and 22SH environments and the combined BLUEs. The scanning step was set to 1.0 cM, and background markers were selected by stepwise regression with a probability threshold (PIN) of 0.001. The significance threshold for the logarithm of odds (LOD) score was determined by 1000 permutation tests at a genome-wide significance level of α=0.05 [40]. QTL support intervals were defined using the one-LOD drop criterion. The proportion of phenotypic variation explained (PVE) was estimated for each detected QTL. QTL-by-environment interactions were not formally evaluated using a multi-environment QTL model. QTLs were named according to the format “q + trait abbreviation + chromosome number + locus number ”, and repeated detections of the same QTL across datasets were assigned the same designation. To facilitate subsequent regional association analysis and candidate gene identification, the physical intervals were converted from the SHL01 reference genome to B73 RefGen_v4 coordinates by aligning the flanking sequences of the QTL intervals to the B73 reference genome (Table S2).

### 4.5. Region Association Analysis and Candidate Gene Identification

The genotype and phenotypic data used for candidate-region association analysis were obtained from a previously established sweet and waxy maize association panel developed by our research group [28]. The panel comprised 159 inbred sweet corn lines with available HKW records. Among these lines, 150 with complete phenotypic records for HKW, KL, and KW were included in the candidate-region association analysis. The remaining nine lines had HKW records but lacked KL and/or KW records. These lines were excluded from the three-trait association analysis but were retained for HKW haplotype analysis when valid genotype information was available. Sample identifiers, phenotype availability, and analysis inclusion status are provided in Supplementary Table S6.

Genotype information was obtained from the original resequencing dataset. SNP markers located within the major QTL intervals identified by linkage mapping were extracted using PLINK v1.90 [41]. The four regional datasets initially contained 916, 287, 405, and 390 SNPs, respectively. GEMMA included 882, 271, 346, and 373 SNPs, respectively, in the corresponding association analyses. Regional association analysis was performed using GEMMA v0.98.5 based on a linear mixed model (LMM), expressed as:

(4) y=Xβ+Zu+ε

where *y* represents the phenotypic values, Xβ represents fixed effects, Zu represents random genetic effects, and ε represents residual errors. Association testing was performed using GEMMA v0.98.5 with the -lmm 1 option. To further evaluate population structure, PCA was performed using 82,657 LD-pruned genome-wide SNP markers from the 150 association lines. In addition, leave-one-chromosome-out (LOCO) kinship matrices were constructed using genome-wide markers excluding chromosome 2 or 7, depending on the region analyzed. Three models were compared using the original regional SNP datasets: the region-specific kinship model without PCs (M1), the LOCO kinship model without PCs (M2), and the LOCO kinship model incorporating the first three PCs (M3). For M1, a standardized kinship matrix was calculated using the regional SNP markers with the GEMMA -gk 2 option. For M2 and M3, LOCO kinship matrices were calculated using genome-wide LD-pruned SNP markers after excluding the corresponding tested chromosome. Model calibration was assessed using Q–Q plots and genomic inflation factors ($\lambda_{\mathrm{GC}}$). Manhattan and Q–Q plots were generated using the CMplot package (version 4.5.1) in R (version 4.4.2). The CMplot package is available at https://github.com/YinLiLin/CMplot (accessed on 29 October 2026). A suggestive threshold of p<1/n was used to screen SNP–trait associations, where *n* represents the number of SNP markers tested within each QTL region. To further evaluate the association signals, *p*-values from the five regional association analyses were adjusted using the Benjamini–Hochberg false discovery rate (FDR) procedure and Bonferroni correction. All SNP–trait tests across the five analyses were treated as one family of tests, with adjusted p<0.05 considered significant. SNP effect estimates (β), standard errors (SEs), allele frequencies (AFs), and Wald test *p*-values were obtained from the GEMMA association results. LD analysis was performed around the selected association signals using LDBlockShow (v1.40) [42]. Pairwise LD between SNPs was evaluated using the squared correlation coefficient ($r^{2}$) with the -SeleVar 2 option in LDBlockShow. Local haplotype blocks were identified using the Gabriel et al. method (-BlockType 1) to characterize the LD patterns within the selected genomic regions. Genes located within or near these regions were identified based on the B73 RefGen_v4 genome annotation. Potential candidate genes were prioritized according to their physical positions relative to the associated SNPs, local LD patterns, and functional annotations. The selected genes were further examined using publicly available transcriptomic data and haplotype analysis.

### 4.6. Expression Analysis of Candidate Genes

The developmental expression patterns of the candidate genes were examined using the publicly available B73 embryo and endosperm transcriptome dataset E-MTAB-4395 [20]. The FPKM values were transformed using $\log_{2}\left(\mathrm{FPKM}+1\right)$, followed by row-wise min–max scaling to the range of −2 to 2 for heatmap visualization. The scaling was applied separately to each gene to show relative changes across developmental stages. Gene identifiers and the corresponding FPKM values are provided in Table S5.

### 4.7. Haplotype Analysis

Haplotype analysis was performed for *Zm00001d020325* and *Zm00001d020323* using all 159 lines with available HKW phenotypic data. This set includes the 150 lines used for regional association analysis plus 9 additional lines that had HKW records but lacked KL and/or KW records. For *Zm00001d020323*, polymorphic markers within the gene and its flanking 2-kb regions were screened, and a total of 23 polymorphic markers were identified. To control for multiple testing, the Bonferroni-corrected significance threshold was set at (0.05/23). Significant markers were further evaluated by comparing phenotypic differences among their genotype classes. SNPs showing significant associations and clear differences in phenotypic values among genotype classes were selected for haplotype construction. Individuals carrying the major haplotypes were grouped according to their allelic combinations, and haplotype genotypes were merged with HKW phenotypic data. Differences in HKW among haplotypes were evaluated using one-way analysis of variance (ANOVA), and the Kruskal–Wallis test was additionally performed as a non-parametric test. Multiple comparisons among haplotype groups were conducted at (*p* < 0.05).

## 5. Conclusions

In this study, an integrated genetic and genomic approach was used to investigate the genetic basis of kernel size and weight-related traits in sweet corn. The identified QTLs revealed substantial genetic variation for HKW, KL, and KW, with chromosome 7 emerging as a particularly important genomic region containing multiple loci for kernel-related traits. Candidate-region association analysis further characterized the selected QTL regions and highlighted several potential candidate genes associated with kernel-related traits. Among these, *Zm00001d020325* remained a potential candidate based on its functional annotation and available expression evidence. Haplotype analysis of the neighboring *Zm00001d020323* revealed differences in HKW among haplotype groups, although these findings remain exploratory and require further validation. Together, these results contribute to a better understanding of the genetic architecture of kernel-related traits in sweet corn and provide candidate genes and genomic regions for future functional validation and genetic improvement.

## Figures and Tables

**Table 1:** Descriptive statistics, analysis of variance, and broad-sense heritability of kernel-related traits in the RIL population across two environments.

Environment	Parameter	HKW (g)	KL (mm)	KW (mm)
21SH	Range	9.80–20.96	10.12–13.72	7.68–11.09
	Mean ± SE	14.08 ± 0.14	11.77 ± 0.05	9.12 ± 0.04
	CV (%)	14.13	6.63	6.58
	Skewness	0.55	0.23	0.27
	Kurtosis	0.49	−0.43	0.11
22SH	Range	4.70–16.36	7.16–12.96	5.20–10.08
	Mean ± SE	10.44 ± 0.15	10.14 ± 0.08	7.85 ± 0.07
	CV (%)	19.35	10.45	11.97
	Skewness	0.12	0.05	−0.27
	Kurtosis	0.61	−0.30	−0.15
Genotype	F-value	7.3581 *	9.5928 *	7.2165 *
Genotype × Environment	F-value	3.6148 *	5.8944 *	5.5237 *
$H^{2}$		0.64	0.59	0.53

$H^{2}$, broad-sense heritability; HKW, hundred-kernel weight; KL, kernel length; KW, kernel width; CV, coefficient of variation; SE, standard error. 21SH, Shanghai in 2021; 22SH, Shanghai in 2022. Genotype and genotype × environment interaction effects were evaluated by analysis of variance (ANOVA). * indicates significance at p<0.01.

**Table 2:** QTLs identified for grain-related traits in the RIL population across different environments.

Environment	QTL	Chr	Interval	LOD	Add	PVE (%)
21SH	**qKL2-1**	**2**	**1,563,041–2,078,610**	**5.36**	**0.25**	**10.78**
	qKL7-1	7	2,363,335–3,193,579	3.74	−0.21	7.27
	qKW2-1	2	10,571,574–11,061,839	4.18	0.17	8.26
22SH	**qHKW7-1**	**7**	**104,908,152–106,983,248**	**6.37**	**−0.77**	**12.48**
	**qKL7-2**	**7**	**4,800,757–5,344,003**	**6.32**	**−0.44**	**13.90**
	**qKW7-1**	**7**	**4,800,757–5,344,003**	**5.48**	**−0.34**	**13.10**
BLUE	qHKW7-2	7	4,800,757–5,344,003	5.26	−0.62	9.38
	qHKW10-1	10	136,130,642–136,965,507	3.34	−0.47	5.37
	**qKL7-3**	**7**	**3,674,203–4,034,597**	**6.65**	**−0.31**	**13.40**
	qKW6-1	6	83,130,639–84,835,891	3.80	−0.18	4.04
	qKW7-1	7	4,800,757–5,344,003	6.88	−0.26	8.15
	qKW7-2	7	168,962,545–169,257,167	8.62	−0.28	9.76

21SH, Shanghai in 2021; 22SH, Shanghai in 2022; BLUE, best linear unbiased estimate. Add, additive effect; PVE, phenotypic variation explained by each QTL. Bold values indicate QTL detections with an estimated PVE > 10%, classified as major-effect QTLs under the operational criterion used in this study.

**Table 3:** Candidate genes identified within QTL regions associated with kernel traits.

QTL	Chr	Candidate Gene	Position (bp)	Functional Annotation
qKL2-1	2	*Zm00001d001845*	1,718,904–1,720,502	Unknown function
qKL7-2	7	*Zm00001d018770*	4,764,934–4,775,821	GYF domain-containing protein
qKL7-3	7	*Zm00001d018732*	3,337,458–3,344,906	Chaperone DnaJ-domain superfamily protein
qHKW7-1	7	*Zm00001d020323*	106,543,662–106,547,885	Unknown function
	7	*Zm00001d020325*	106,753,872–106,761,586	potassium high-affinity transporter 23 (HAK23)

## Data Availability

The original contributions presented in this study are included in the article. Further inquiries can be directed to the corresponding authors.

## Supplementary Materials

The following supporting information can be downloaded at https://www.mdpi.com/article/10.3390/ijms27219863/s1.

## Abbreviations

The following abbreviations are used in this manuscript:

QTL	Quantitative Trait Locus/Quantitative Trait Loci
KL	Kernel Length
KW	Kernel Width
HKW	Hundred-Kernel Weight
RIL	Recombinant Inbred Line
PVE	Phenotypic Variance Explained
MAF	Minor Allele Frequency
LOD	Logarithm of Odds
LMM	Linear Mixed Model
LD	Linkage Disequilibrium
BLUE	Best Linear Unbiased Estimate
GBTS	Genotyping by Target Sequencing
