# Supplementary material for: Integrating QTL Mapping and Candidate-Region Association to Investigate Kernel Size and Weight in Sweet Corn
Source: Int J Mol Sci. 2026 Nov 3;27(21):9863. doi: 10.3390/ijms27219863 (PMC13763696; doi:10.3390/ijms27219863)
Supplement: Supplementary file 1 [file ijms-27-09863-s001.zip › Supplementary Figures.pdf]

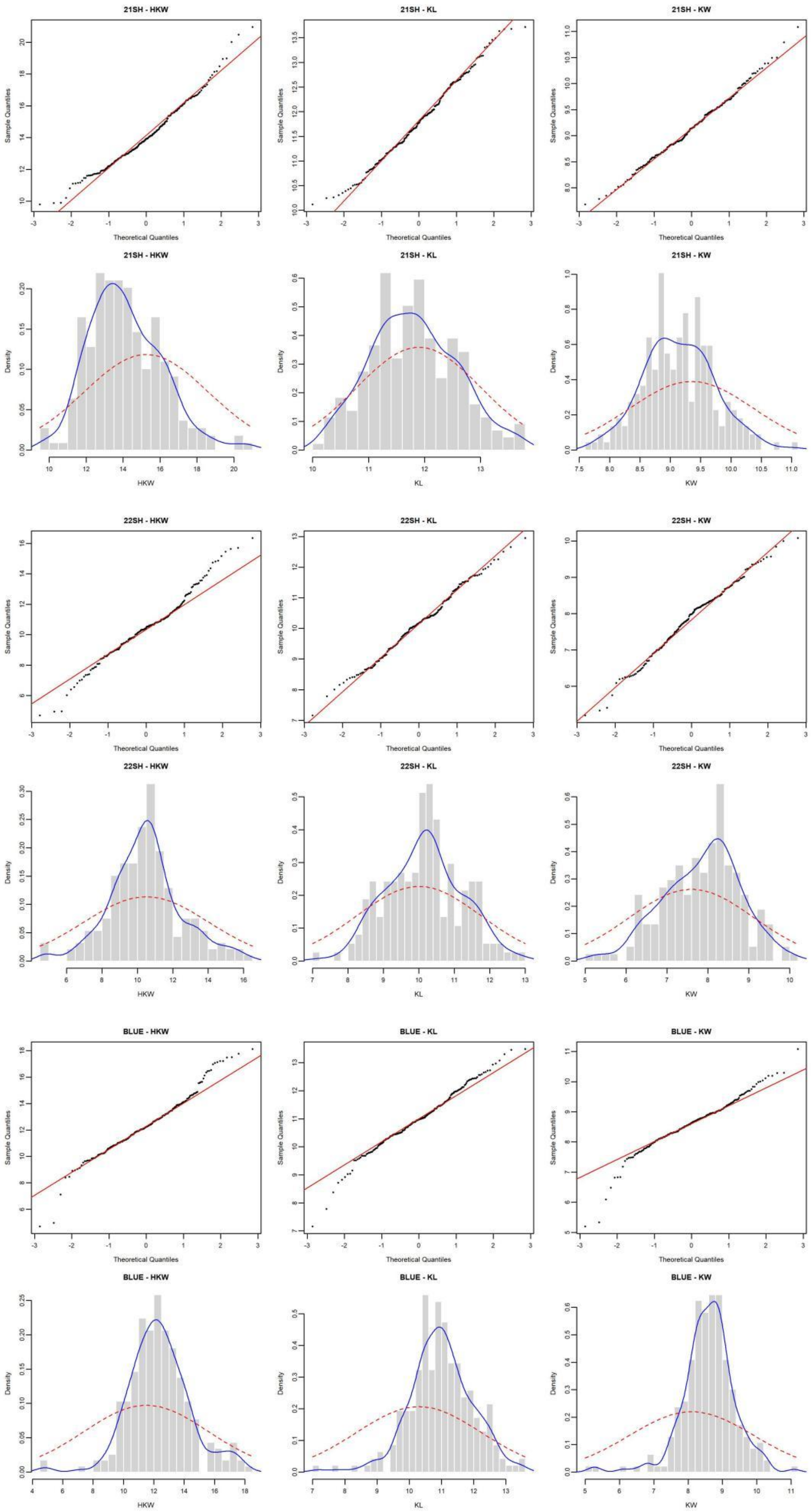

Figure S1 Distribution and normality assessment for kernel size and weight under different environments.

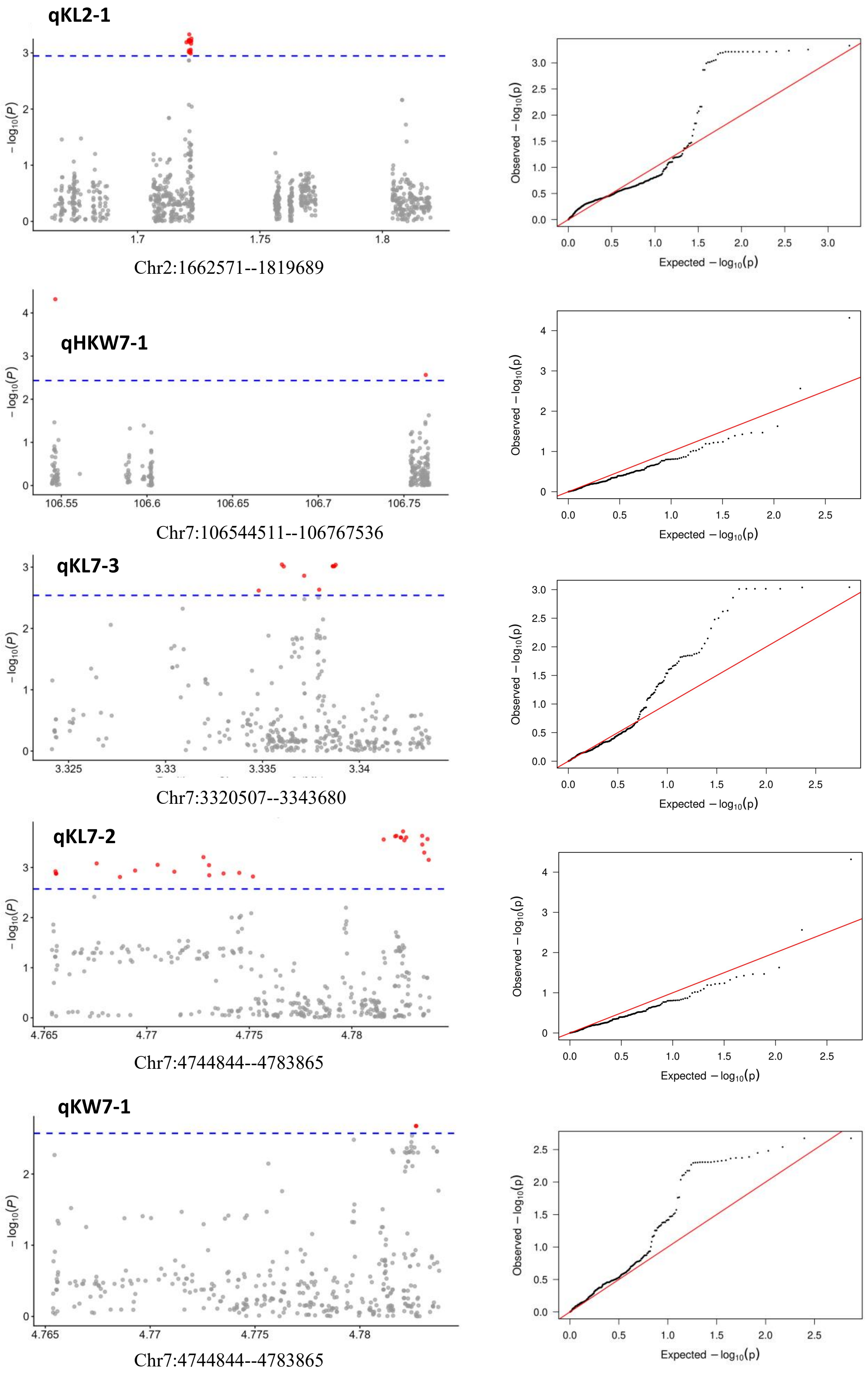

Figure S2 Regional Manhattan and Q--Q plots for the five QTL analyses. The horizontal dashed lines indicate the suggestive threshold of $P < 1/n$, where n is the number of SNPs tested within each region.

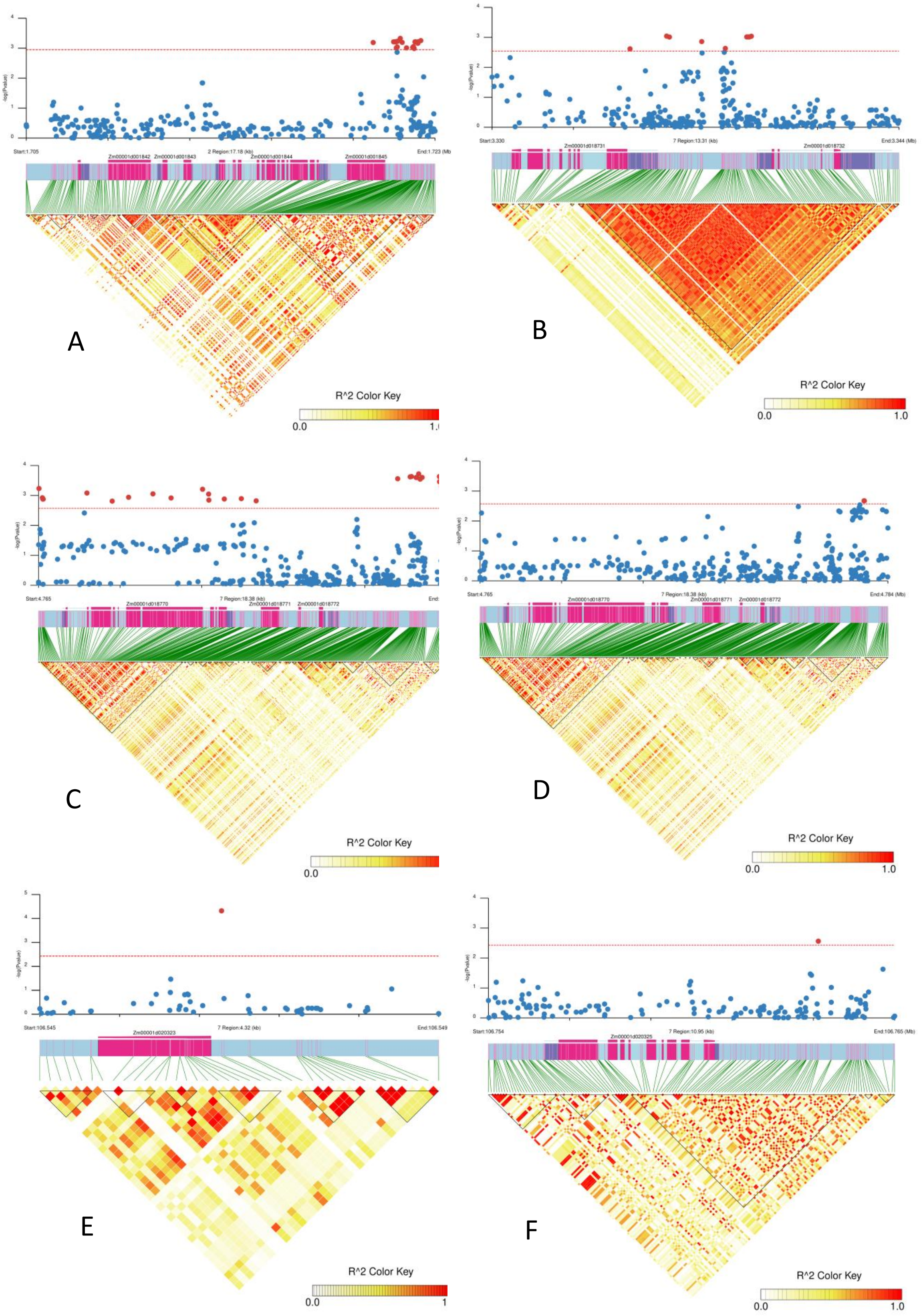

Figure S3. Local linkage disequilibrium (LD) patterns and haplotype block structures within the selected QTL regions. (A) *qKL2-1*; **(B)** *qKL7-3*; **(C)** *qKL7-2*; **(D)** *qKW7-1*; **(E,F)** two regions examined within *qHKW7-1*.

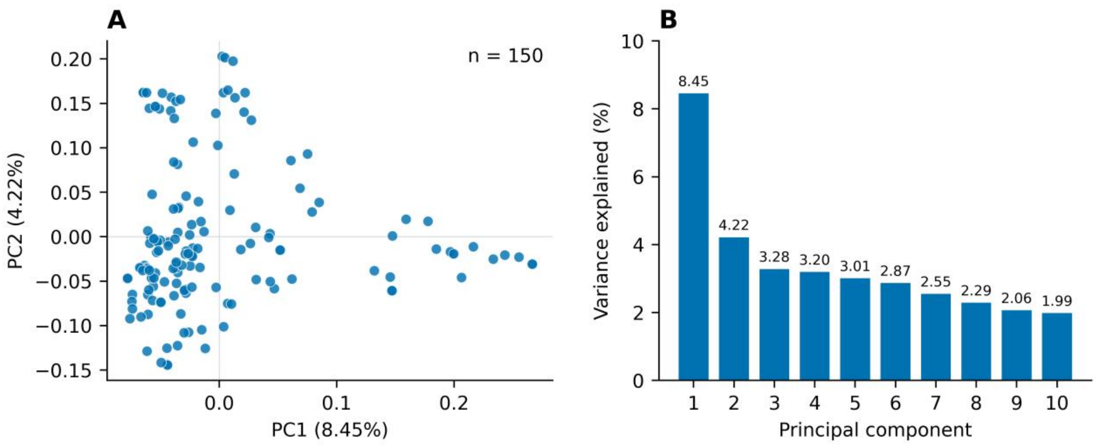

Figure S4. Population structure of the sweet corn association panel. (A) Principal component analysis (PCA) of 150 sweet corn lines based on 82,657 LD-pruned genome-wide SNP markers. PC1 and PC2 explained 8.45% and 4.22% of the total genetic variation, respectively**. (B)** Percentage of genetic variation explained by the first ten principal components.

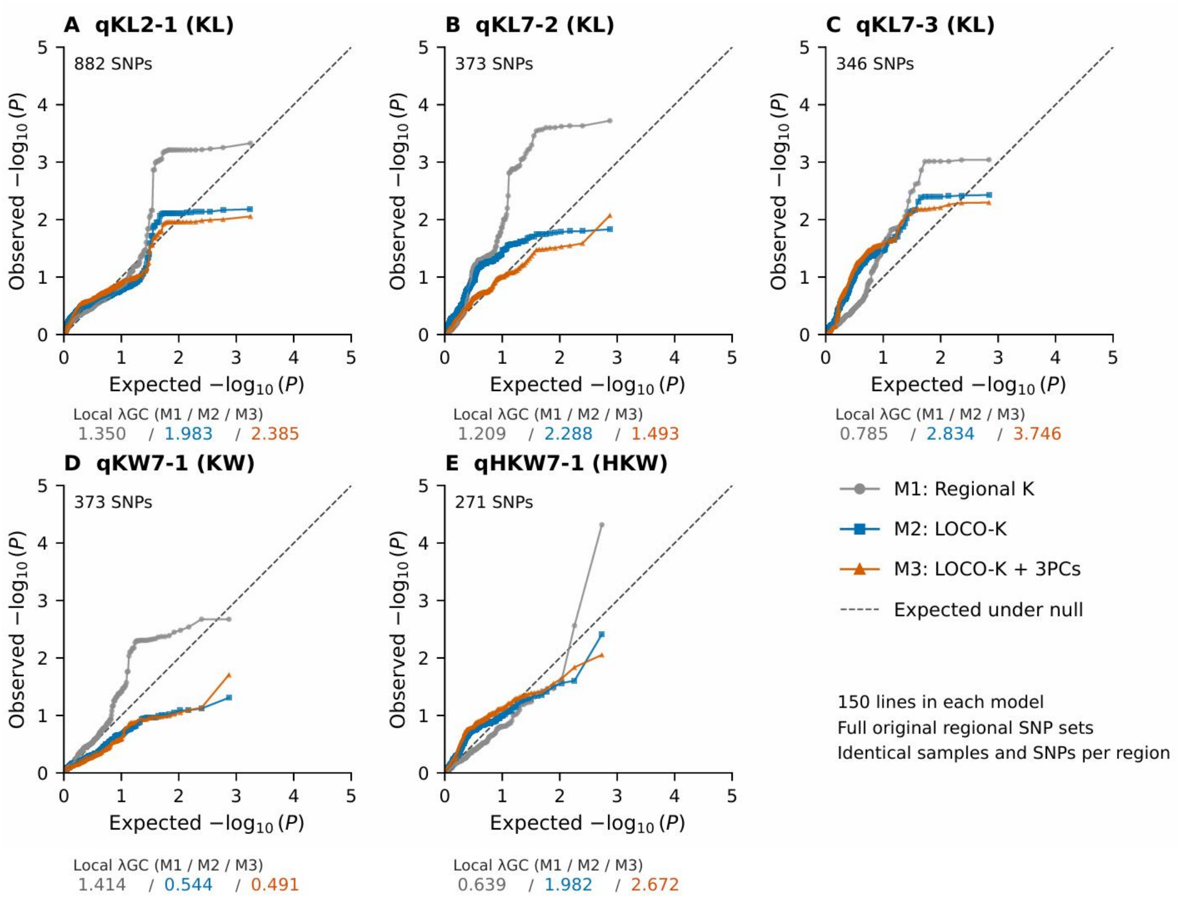

Figure S5. Q–Q plots comparing three regional association models. Panels **A–E** represent qKL2-1 (KL, 882 SNPs), qKL7-2 (KL, 373 SNPs), qKL7-3 (KL, 346 SNPs), qKW7-1 (KW, 373 SNPs), and qHKW7-1 (HKW, 271 SNPs), respectively. M1, regional kinship model; M2, leave-one-chromosome-out (LOCO) kinship model; M3, LOCO model with PC1–PC3. All models used the same 150 lines and regional SNPs. Dashed lines indicate the null expectation, while color-coded values indicate local genomic inflation factors (λGC), which do not represent genome-wide calibration.
